# Evaluation of the Impact of Chlorhexidine Mouth Rinse on the Bond Strength of Polycarbonate Orthodontic Brackets: A Case-Control Study

**DOI:** 10.7759/cureus.38227

**Published:** 2023-04-27

**Authors:** Jaideep Singh, Amit Kumar, Ekta Gupta, K. Shiva Charan Yadav, Gajji Renuka, Vani Singh, Anushree Tiwari, Ramanpal Singh

**Affiliations:** 1 Department of Orthodontics and Dentofacial Orthopaedics, Maharana Pratap Dental College, Kanpur, IND; 2 Department of Orthodontics and Dentofacial Orthopaedics, Dr. B.R. Ambedkar Institute of Dental Sciences and Hospital, Patna, IND; 3 Department of Orthodontics and Dentofacial Orthopaedics, Siddhpur Dental College and Hospital, Gujarat, IND; 4 Department of Periodontology, Malak Al Rahma Polyclinic, Riyadh, SAU; 5 Department of Orthodontics, Malak Al Rahma Polyclinic, Riyadh, SAU; 6 Public Health Dentistry, Bhabha College of Dental Sciences, Bhopal, IND; 7 Clinical Quality and Value, American Academy of Orthopaedic Surgeons, Rosemont, USA; 8 Oral Medicine and Radiology, New Horizon Dental College and Research Institute, Chhattisgarh, IND

**Keywords:** orthodontics, brackets, shear bond strength, polycarbonate brackets, chlorhexidine

## Abstract

Background: Shear bond analysis is the procedure used most frequently to gauge the tensile strength of adhesives incorporated in orthodontic treatments. In shear tensile strength analysis, pressure is placed as close as feasible to the interface between the orthodontic bracket and the surface of the tooth, parallel to the long axis of the tooth. Although numerous research on extracted teeth of human and bovine teeth have been conducted, there may still be variables such as pH, humidity, temperature, and others that could affect how these materials behave in the mouth cavity. The impact of chlorhexidine (CHX) on the binding capacity for non-metallic orthodontic brackets in vivo is not well understood.

Objective: The goal of the current study is to determine how mouth rinses containing 0.12% CHX affect the adhesive strength of polycarbonate orthodontic brackets.

Methods and Materials: Thirty-four patients were part of the test category, and they were instructed to wash their oral cavity for approximately 30 seconds using 20 ml of 0.12% CHX gluconate (Septodent). Thirty-four patients made up the control category and were instructed to wash their oral cavity for 30 seconds with a placebo mouthwash of a similar hue (20 ml). Both types of mouthwash were administered to the participants by an administrator who was not specifically involved in the trial and were kept in 120 ml labeled plastic bottles. The study participants were also kept unaware of the type of mouthwash. For the mouthwash utilized by study participants, a double-blinding technique was applied.

Results: Thirty-four patients were evaluated in the test category. Since the orthodontic bracket broke in two patients, therefore, 32 patients were evaluated in the control category. The mean value of the strength of the shear bond in the experimental category was 15.32 megapascal (Mpa). The SD value was 2.51. The mean value of the strength of the shear bond in the control subgroup was 15.63. On statistical analysis, the t-value was 0.47. The p-value was 0.671. The difference in findings of the strength of the shear bond was statistically non-significant.

Conclusion: The results of this investigation allow us to draw the conclusion that the shear bond properties of polycarbonate orthodontic brackets are unaffected when treated with 0.12% CHX preceding the binding. The clinically meaningful adhesion strength was likewise attained by the polycarbonate orthodontic brackets.

## Introduction

Orthodontic treatment involving fixed appliances alters the oral setting in a manner that encourages the buildup of periodontal plaque around the orthodontic brackets, which can cause periodontal issues, dental caries, and white spot defects [1,2]. To minimize caries and gingival inflammation while receiving orthodontic treatment, a variety of preventative strategies have been documented in the scientific literature [3]. Several substances have been tried to reduce the incidence of caries as well as decalcification in patients receiving orthodontic therapy. Antimicrobial drugs are typically recommended in addition to traditional therapy to decrease the deposition of periodontal plaque biofilm and enamel decalcification during therapy [4-9].

Among the most often utilized antibacterial substances in dental care is chlorhexidine (CHX). It has been demonstrated to be successful in preventing plaque buildup and treating periodontal disorders in conjunction [8-12]. Although coating the tooth structure with CHX can improve antimicrobial defense, it may negatively impact the strength of the bond between teeth and orthodontic brackets [13]. These consequences have been studied using sealants, primers, varnishes, and toothpaste incorporating CHX along with basic oral prophylaxis [14]. In addition, the impact of applying several CHX preparations on etched tooth enamel prior to bonding was previously evaluated [15]. These in vitro experiments revealed conflicting results that suggested using CHX on the tooth enamel prior to bonding could reduce the binding affinity of metal orthodontic brackets [16].

In order for an orthodontic bracket to be deemed practically viable, it must be able to withstand loads between 5.9 MPa and 7.8 MPa [15]. The shear bond analysis is the procedure used most frequently to gauge the tensile strength of adhesives incorporated in orthodontic treatments. In shear tensile strength analysis, pressure is placed as close as feasible to the interface between the orthodontic bracket and the surface of the tooth, parallel to the long axis of the tooth. Although numerous research on extracted teeth of human and bovine teeth have been conducted, there may still be variables such as pH, humidity, temperature, and others that could affect how these materials behave in the mouth cavity [17-22]. The impact of CHX on the binding capacity for non-metallic orthodontic brackets in vivo is not well understood. Therefore, the goal of the current study is to determine how mouth rinses containing 0.12% CHX affect the adhesive of polycarbonate orthodontic brackets. With the help of a universal testing machine, the load needed to eliminate the orthodontic brackets was calculated in order to determine the strength of the shear bond.

## Materials and methods

The study was approved by the Maharana Pratap Dental College ethics committee with number IEC/2017/23 The research consisted of orthodontic clients who had at least two first premolars planned for prophylactic extraction as an aspect of their therapy, and the study participants who were not suffering from any systemic diseases were included in the study. Exclusion criteria include the patients who were found to have a history of use of any mouthwash and the patients who didn’t give their informed consent.

Patients were informed of the trial's specifics, and all volunteers and their parents' signed consents were obtained before the research could begin. Every subject was recommended for a mouth prophylaxis regimen, which included using a commercial mouthwash for seven days prior to bonding. The patients were not asked to visit the clinic every day for seven days. They were given instructions only at once. A matched pairs design was used to structure the research, with one category receiving a test mouthwash and the other serving as a control category.

Employing tables containing random numbers, the volunteers were divided into the two categories at random in accordance with a previously established procedure. The sample size was determined by using the formula n = (z)2 p ( 1 - p ) / d2 where n = sample size, z = level of confidence according to the standard normal distribution (for a level of confidence of 95%, z = 1.67, p = estimated proportion of the population that presents the characteristic (when unknown we use p = 0.5), d = tolerated margin of error (for example we want to know the real proportion within 5%). Using the above formula, a minimum sample size of 33.4 equivalent to 34 was calculated.

Thirty-four patients were part of the test category, and they were instructed to wash their oral cavity for approximately 30 seconds using 20 ml of 0.12% CHX gluconate (Septodent). Thirty-four patients made up the control category and were instructed to wash their oral cavity for 30 seconds with a placebo mouthwash of a similar hue (20 ml). Both types of mouthwash were administered to the participants by an administrator who was not specifically involved in the trial and were kept in 120 ml labeled plastic bottles. The study participants were also kept unaware of the type of mouthwash. For the mouthwash utilized by study participants, a double-blinding technique was applied.

With the exception of the first permanent premolars that were scheduled for orthodontic extraction, directly attached conventional edgewise stainless steel orthodontic brackets (0.022 inches by 0.028 inches slots) were attached on each tooth after a one-week break, and molar orthodontic bands were bonded for every participant included in the research across both categories. The first premolars were attached with a directly attached standard edgewise polycarbonate orthodontic bracket (0.022 inches by 0.028 inches slot). Every tooth's outer layer was touched up for one minute with water as well as pumice, followed by rinsing and drying. A 37% phosphoric acid in gel form was then used to erode the tooth enamel for 30 seconds.

All orthodontic brackets were attached using a light-cured (Ivoclar Vivadent Te-Econom, India) bonding agent in accordance with the manufacturer's guidelines after acid etching. After applying constant force to fully secure the orthodontic brackets to the teeth, an extra bonding agent was scraped off. Then, using light-curing equipment for 40 seconds, each orthodontic bracket was exposed to 10 seconds of light for its occlusal surfaces, mesial surface, gingival surface, and gingival surface. The first permanent premolars were extracted and then rinsed and cleared of any remnant of soft tissue utilizing curettes and kept in a liquid of 0.1% thymol 28 days after the brackets were bonded. The brackets were placed before extraction. The extraction was carried out after 28 days of bonding. Proper care was taken during extraction to avoid damage to brackets. Extractions were carried out by experienced oral and maxillofacial surgeons. The samples were affixed using quick-setting acrylic resin in specially constructed Teflon rings that were 10 mm in diameter and 15 mm high. A digital caliper was used to determine the overall area of the bracket foundation, which came out to 13.63 mm^2^.

**Figure 1:**
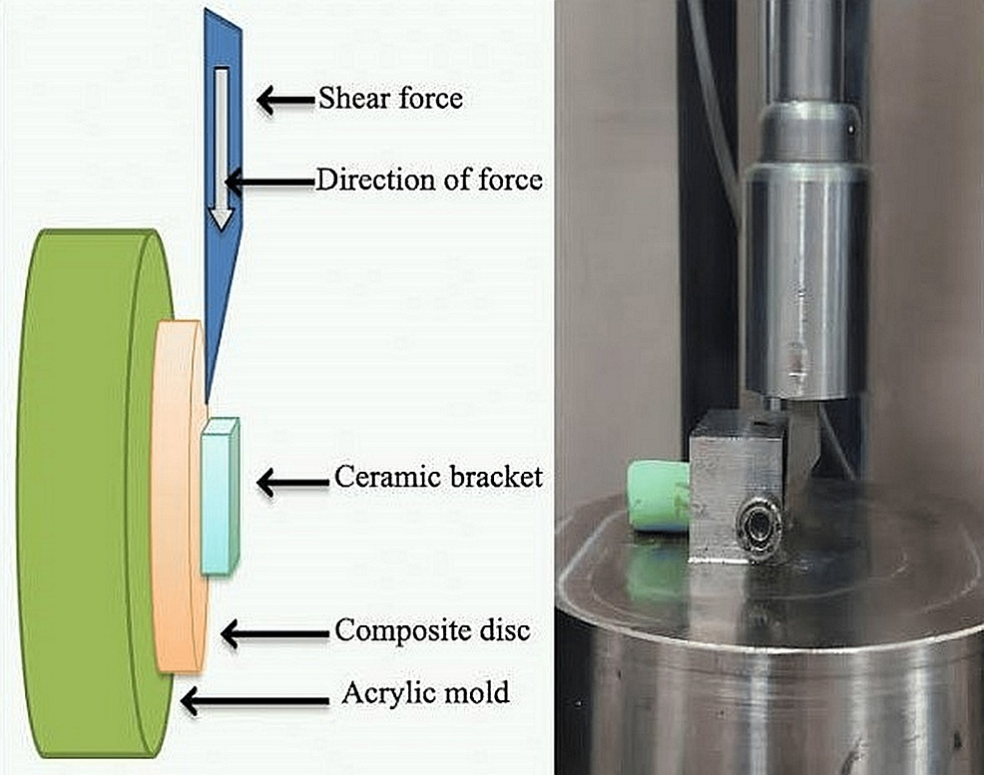
Schematic as well as lab image of the procedure

A load was applied to the orthodontic bracket using an Instron universal testing apparatus marketed by Lloyd Instruments, UK, which resulted in tensile stresses at the interface of the tooth and orthodontic bracket. The outcomes of each assessment were documented on a computer attached to the apparatus (Nexygen software). A crosshead rate of 0.5 mm/min was used to determine the shear adhesion strength. The strength of the shear bond has been measured in megapascals (MPa) by dividing the greatest force necessary to remove the orthodontic brackets by the base area of the orthodontic brackets (1 MPa = 1 N/mm2) (Figure 1).

The SPSS Statistics Version 29 (IBM Corp. Released 2022. IBM SPSS Statistics for Windows, Version 29.0. Armonk, NY: IBM Corp) was used to statistically evaluate the data. The Shapiro-Wilk method was used to assess the bond resilience of the composite adhesion for normalcy, and it was discovered that the information had a normal distribution with homogeneous variation between subgroups. Employing parametric statistics, the statistical analysis of the strength of the bond was carried out. The shear strength of bonds between the two categories was contrasted using Student's t-test after descriptive statistics for each category were computed. P ≤ 0.05 was used to evaluate significance.

## Results

The mean age of the study participants in the test category was 22.52 ± 1.3 years, while it was 22.36 ± 1.4 years in the control category. About 38.9% of the study participants in the test category were males, while 41.7% of the study participants were males in the control category. The duration of treatment in the control group was 24±1.2 months, while the duration of treatment in the control subgroup was 23 ± 1.6 months. The difference in findings in both subgroups was statistically nonsignificant (p =0.87) (Table 1).

**Table 1:** Demographic details of the study participants in both test and control categories

	Test category (n = 34)	Control category (n = 32)
Mean age ± SD	22.52 ± 1.3 years	22.36 ± 1.4 years
Male (%)	38.9	41.7
Duration of treatment in months (mean ± SD)	24 ± 1.2	23 ± 1.6
t-value	0.48	
p-value	0.87	

Thirty-four patients were evaluated in the test category. Since the orthodontic bracket broke in two patients, therefore, 32 patients were evaluated in the control category. The mean value of the strength of the shear bond in the experimental category was 15.32 Mpa. The SD value was 2.51. The mean value of the strength of the shear bond in the control subgroup was 15.63. The SD value was 2.47. On the statistical analysis, the t-value was 0.47. The p-value was 0.671. The difference in findings of the strength of the shear bond was statistically non-significant (Table 2).

**Table 2:** Descriptive statistics of shear bond strengths (in MPa) of the two categories

	Test category (n = 34)	Control category (n = 32)
Mean	15.32	15.63
SD	2.51	2.47
t-value	0.47	
p-value	0.671	

## Discussion

The purpose of the study was to evaluate how CHX mouthwash affected the adhesion of polycarbonate orthodontic brackets. We discovered that neither the control category nor the study category showed any discernible differences. The strength of the shear bond was unaffected by the subgroup that applied CHX for seven days before attachment with the polycarbonate. This finding is consistent with past reports by Bishara et al. [13], Damon et al. [16], Demir et al. [18], and Filler et al. [20]. In vitro, experimental frameworks were used for the majority of the previous publications. Due to the differences in the design of experiments and the materials employed, a relevant comparison of the present investigation is not feasible. Although ceramic orthodontic brackets and polycarbonate orthodontic brackets are more attractive, they have some operational and mechanical constraints. A ceramic filler has been added to polycarbonate orthodontic brackets recently in an effort to strengthen them and prevent them from discoloring. Nevertheless, very little is known about how well it performs in clinical settings [23].

For both categories, the average values of bond strength ranged between 11.49 MPa to 18.75 MPa. The adhesion of the orthodontic polycarbonate brackets to the enamel was not affected by CHX in this trial. There are two suggested reasons for this observation. First, any major modifications to the enamel layer caused by CHX may have been undone by the acid etching procedure used in the procedure of bonding [23-29]. A study by Legler et al. on the extent of erosion in the enamel surface brought on by different phosphoric acid treatment durations and concentrations showed that a 30-second exposure to a 37% phosphoric acidic media produced an etch area of about 16 µm [30]. This means that if CHX permeated enamel to this degree or less, the etching may have eliminated it. Second, due to the molecular relative position and the fact that CHX particles are considerably bigger than fluoride ions as well as hydroxyapatite crystals, CHX cannot alter the surface of the enamel [30,31].

The findings of the present investigation can be explained by either the absence of CHX effects or acid etching, which destroyed the damaged surface enamel and left an untouched foundation for adhesion. Contradictory results have been reported by certain research, including a reduction in the interfacial adhesion of the Transbond composite once various CHX formulations were placed in vitro as a coating over a scratched enamel surface and precisely before bonding of an orthodontic bracket. Strength and durability levels were discovered to be insufficient for clinical use [13-21]. These investigations, however, used various CHX forms, bracket types, and bonding techniques on a scratched enamel, as well as various methodological approaches, which may account for the discrepancy in the results. The strength of this study was its in vivo nature. Most of the previous studies were conducted in in vitro settings.

The study limitations include incorporating a CHX mouth rinse may be helpful in reducing bacterial biofilm, but there have been reports of a number of negative side effects, along with a bitter aftertaste, an elevation in calculus buildup, and brown staining of the teeth as well as the tongue [32]. Before using CHX mouthwash in routine clinical practice, additional randomized controlled trials are required to assess the effects of continued usage of CHX on shear strength properties, color durability of polycarbonate orthodontic brackets, and possible negative reactions completely warranted.

## Conclusions

The results of this investigation allow us to draw the conclusion that the shear bond properties of polycarbonate orthodontic brackets are unaffected when treated with 0.12% CHX preceding the binding. The clinically meaningful adhesion strength was likewise attained by the polycarbonate orthodontic brackets.
